# Overly Strong Priors for Socially Meaningful Visual Signals Are Linked to Psychosis Proneness in Healthy Individuals

**DOI:** 10.3389/fpsyg.2021.583637

**Published:** 2021-04-08

**Authors:** Heiner Stuke, Elisabeth Kress, Veith Andreas Weilnhammer, Philipp Sterzer, Katharina Schmack

**Affiliations:** ^1^Department of Psychiatry and Psychotherapy, Charité – Universitätsmedizin Berlin, Berlin, Germany; ^2^Bernstein Center of Computational Neuroscience, Berlin, Germany

**Keywords:** face processing, perceptual bias, predictive coding, psychosis proneness, hallucination, gaze detection

## Abstract

According to the predictive coding theory of psychosis, hallucinations and delusions are explained by an overweighing of high-level prior expectations relative to sensory information that leads to false perceptions of meaningful signals. However, it is currently unclear whether the hypothesized overweighing of priors (1) represents a pervasive alteration that extends to the visual modality and (2) takes already effect at early automatic processing stages. Here, we addressed these questions by studying visual perception of socially meaningful stimuli in healthy individuals with varying degrees of psychosis proneness (*n* = 39). In a first task, we quantified participants’ prior for detecting faces in visual noise using a Bayesian decision model. In a second task, we measured participants’ prior for detecting direct gaze stimuli that were rendered invisible by continuous flash suppression. We found that the prior for detecting faces in noise correlated with hallucination proneness (*r* = 0.50, *p* = 0.001, Bayes factor 1/20.1) as well as delusion proneness (*r* = 0.46, *p* = 0.003, BF 1/9.4). The prior for detecting invisible direct gaze was significantly associated with hallucination proneness (*r* = 0.43, *p* = 0.009, BF 1/3.8) but not conclusively with delusion proneness (*r* = 0.30, *p* = 0.079, BF 1.7). Our results provide evidence for the idea that overly strong high-level priors for automatically detecting socially meaningful stimuli might constitute a processing alteration in psychosis.

## Introduction

Schizophrenia is characterized by psychotic symptoms such as delusions and hallucinations. Neurocognitive theories that draw on predictive coding and Bayesian theories of brain function have proposed an imbalance between prior expectations and current sensory information as a central disturbance underlying psychotic experiences (Fletcher and Frith, 2009; Adams et al., 2013; Sterzer et al., 2018). In this context, an overly strong prior for socially meaningful signals can account for hallucinatory experiences, such as hearing voices in the absence of causative stimulus, or delusional experiences, such as the feeling of being looked at by strangers (Corlett et al., 2009, 2019).

Consistent with this theoretical framework, an increased tendency to perceive voices in auditory noise has been observed in psychosis and related conditions (Bentall and Slade, 1985; Hoffman et al., 2007; Vercammen et al., 2008; Galdos et al., 2011; Alderson-Day et al., 2017), in line with the idea of overly strong prior for socially meaningful signals in the auditory domain. A similar shift toward perceiving abstract signals, such as pure tones, in auditory noise (Powers et al., 2017) points to the possibility that overly strong priors might affect auditory perception in general.

Hence, while there is evidence to support the idea of overly strong priors for meaningful auditory signals in psychosis, it is currently unclear whether this reflects a generic processing deficits that reliably extends to the visual modality. A few studies have related an increased tendency to perceive faces in visual noise (Partos et al., 2016), and an increased tendency to perceive visual gaze as direct (Rosse et al., 1994; Hooker and Park, 2005; Tso et al., 2012) to psychosis and related conditions, but results have been mixed (see Franck et al., 2002 for a negative report). Assessing relationships between psychotic experiences and the use of priors toward meaningful visual signals is crucial for probing the generalizability of strong prior accounts of psychosis. Here, we therefore related psychosis proneness in individuals from the general population to behavior in a visual detection-in-noise task. We hypothesized that psychosis proneness would positively correlate with the tendency to detect faces in visual noise, and hence a prior toward detecting meaningful stimuli.

Moreover, it is currently unclear that which stage of information processing is affected by overly strong priors underlying psychotic experiences. It is conceivable that overly strong priors might only affect the late, conscious processing stage of cognitive interpretation. Alternatively, the effects of overly strong priors might extend to early, automatic sensory processing stages that determine the access of stimuli to awareness. In the visual domain, the potency of visual stimuli to gain access to awareness can be assessed with interocular masking techniques such as continuous flash suppression (CFS; Tsuchiya and Koch, 2005). In CFS, one eye is presented with a target stimulus, while the other eye is presented with a dynamic mask that initially suppresses the target stimulus from conscious perception. The time that the suppressed stimulus takes to overcome interocular suppression has been proposed as a measure for the potency of a specific stimulus to gain access to awareness (Jiang et al., 2007; Stein and Sterzer, 2014). For example, this “breaking CFS” paradigm (b-CFS; Stein et al., 2011a) has been used to show that suppression times are decreased for stimuli with direct gaze as compared to stimuli with averted gaze (Stein et al., 2011b). Inter-individual variability in breakthrough time depends on individual factors related to the stimuli that compete for perceptual dominance. For example, the advantage for faces with direct gaze in gaining access to awareness is reduced in individuals with autistic traits (Akechi et al., 2014; Madipakkam et al., 2019). Similarly, suppression times are reduced for sad faces in patients with major depression (Sterzer et al., 2011) and for spider stimuli in individuals with spider phobia (Schmack et al., 2016). Here, we asked whether a strong prior for direct gaze may affect those processing stages that determine access of face stimuli to awareness and therefore tested whether suppression times for direct compared to averted gaze may be shorter in individuals with high psychosis proneness.

The “Psychosis Continuum” view postulates that the clinical manifestations of psychosis represent the most extreme form of psychosis proneness, which is continuously distributed in the general population (Barrantes-Vidal et al., 2015; DeRosse and Karlsgodt, 2015). Indeed, psychotic experiences are not confined to clinical populations, but can be found to varying degrees in the general population (Peters et al., 2004; Bell et al., 2006). Interestingly, subclinical psychosis proneness and clinical psychosis are associated with similar risk factors (van Os et al., 2009; Linscott and van Os, 2013) and exhibit a shared factor structure of symptoms (Shevlin et al., 2017). Furthermore, the relatives of patients with psychotic disorders show increased levels of subclinical psychosis proneness, suggesting common genetic underpinnings (Kendler et al., 1993; Fanous et al., 2001; Tienari et al., 2003). Importantly, high levels of subclinical psychosis proneness increase the risk for later clinical psychosis (Chapman et al., 1994; Hanssen et al., 2005; Welham et al., 2009). Taken together, these findings suggest that subclinical and clinical psychotic experiences are mediated by shared processes. Hence, the investigation of subclinical psychosis proneness in non-patient populations can provide insights into the processes underlying psychotic experiences in general, while not being confounded by psychotropic medications or other concomitants of clinical psychotic disorders.

Here, we tested whether delusion and hallucination proneness relate to overly strong priors for detecting socially meaningful stimuli, as quantified in two visual detection tasks. Specifically, we hypothesized that psychosis proneness would correlate to an enhanced prior for detecting faces in noisy sensory information and an enhanced prior for detecting direct gaze in stimuli rendered invisible with continuous flash suppression.

## Materials and Methods

### Participants and Psychometry

Thirty-nine participants were recruited from the general population through advertising. The study was approved by the Ethical Committee of the Charité, Universitätsmedizin Berlin. After complete description of the study to the participants, written informed consent was obtained in accordance with the Declaration of Helsinki of 1975 before participation.

Psychosis proneness was assessed with questionnaires previously validated in non-clinical populations. Here, proneness to delusional ideation was quantified using the Peters Delusion Inventory, 21-item version (PDI-21; Peters et al., 2004). The 21 items of this self-rating questionnaire cover a wide range of delusional convictions including beliefs in the paranormal, grandiosity ideas, or suspicious thoughts. For every endorsed belief, the questionnaire asks for dimensional ratings of belief-related distress, preoccupation, and conviction.

Additionally, proneness to hallucinatory experiences was assessed with the Cardiff anomalous perception scale (CAPS; Bell et al., 2006). This 32-item self-rating scale assesses anomalous perceptual experiences in different sensory domains including proprioception, time perception, somatosensory perception, and visual and auditory perception. The intensity of every anomalous perception is quantified on subscales for intrusiveness, frequency, and distress. As in our previous work (Stuke et al., 2017, 2018), we used total PDI and CAPS scores obtained by adding up their three subscales.

### Face Task

**Figure 1:**
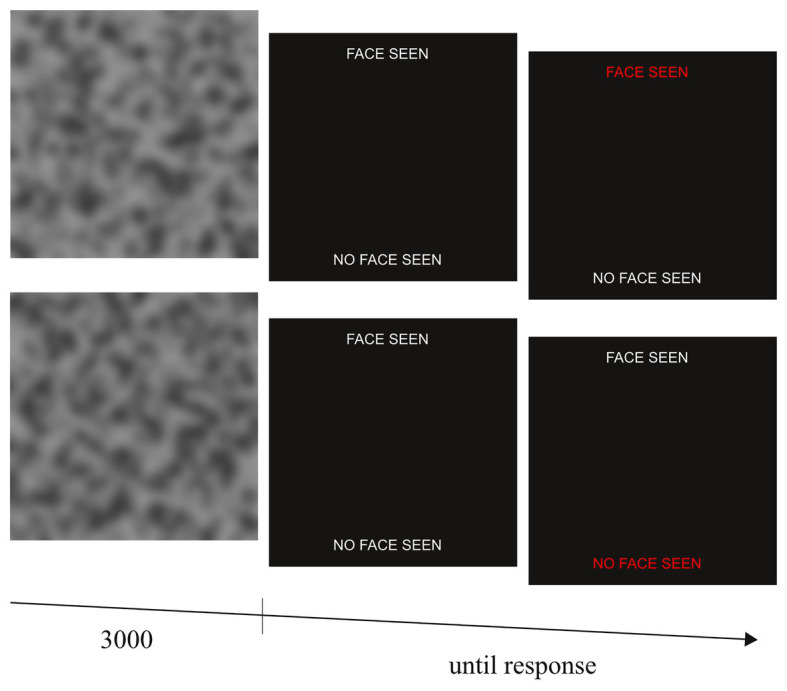
Experimental sequence of the face task for two exemplary trials with (top row) and without (lower row) embedded face. Face images were shown for 3,000 ms followed by a binary forced choice indication of whether a face had been detected by the participants. By fitting a Bayesian model to the individual participants’ behavior, we obtained a measure of the prior for detecting faces.

To quantify priors toward socially meaningful stimuli in visual perception, we measured psychosis-like mispercepts of illusory faces in noise. To this end, we devised a face detection task that required the participants to detect faces embedded in noise. One-hundred stimuli (40 target and 60 noise stimuli) were created. Participants were instructed that a sequence of noisy stimuli will be presented to them and that some of stimuli will contain a human face. Each stimulus was presented for 3,000 ms followed by a forced-choice decision of whether a face was present or not. After a response had been made and a subsequent inter trial interval of 800 ms, the next stimulus was presented (Figure 1).

Stimuli were designed to resemble those that have proven to induce psychosis-like percepts of illusory faces in previous work (Partos et al., 2016). Noise stimuli consisted of a noise pattern only (without embedded face) and were created in three steps using Matlab and the Image Processing Toolbox. Firstly, basic noise patterns were generated by randomly placing a total of 1,000 black circles with diameters varying randomly from 1 to 15 pixels (0.04°–0.64° of visual angle) on a white image of 450 × 450 pixels (19.45° of visual angle). Secondly, the basic noise patterns were degraded by adding multiplicative noise (as implemented in the “speckle” command of the Matlab imnoise routine with a distribution variance of 2). Finally, the resulting noise stimuli were blurred with a Gaussian filter (“gaussian” command of the Matlab imnoise routine with a distribution variance of 10) and image contrast was reduced with the “imadjust” routine (resetting gray scale intensities to values between 0.1 and 0.9). For the target stimuli, 20 adult faces with neutral expression were taken from the Productive Aging Laboratory Face Database (Minear and Park, 2004) and placed at random positions in the noise stimuli before the third step of noise image generation (i.e., before the Gaussian filter and contrast reduction). All faces were oriented upright. The specific image generation parameters were chosen to ensure that participants were imperfectly able to distinguish the faces from the noise stimuli in a pilot study with five participants (discriminability mean = 0.81, SD = 0.03; bias = 1.52, SD = 0.86; computed using signal detection theory equations; Stanislaw and Todorov, 1999).

### Face Task Analysis

Face task behavior was analyzed with a Bayesian model combining an individual prior for detecting faces with a sensory likelihood of a face depending on whether a face was embedded in the stimulus or not.

Hence, the probability of detecting a face in each trial was:

Equation 1:

$$P\left(face\;\;detected\right)=\frac{Prior\times Likelihood}{Prior\times Likelihood+\left(1-Prior\right)\times \left(1-Likelihood\right)}$$

where *Prior* is an estimated free parameter and *Likelihood* was computed as follows.

The sensory likelihood of a face *Likelihood* depended on whether a face was embedded in the stimulus:

Equation 2:

$$Likelihood=Sensitivity^{face}\times {\left(1-Sensitivity\right)}^{1-face}$$

where sensitivity is an estimated free parameter and *face* is a binary vector, indicating whether a face was embedded in each trial.

The objective function to be maximized for each participant was hence:

Equation 3:

$$L=\underset{i=1}{\sum }\log\left(P{\left(face\;\;detected\right)}_{i}^{face\;\;detected_{i}}\times {\left(1-P{\left(face\;\;detected\right)}_{i}\right)}^{\left(1-face\;\;detected_{i}\right)}\right)$$

where *i* is an index denoting the trial number and *face detected* is a binary vector, indicating whether a face was detected in each trial by the participant.

For each participant, this model estimates a prior probability for detecting a face as well as a sensitivity parameter capturing how much the likelihood of detecting a face depended on whether the stimulus contained a face or not. Estimation of individual face prior and sensitivity values by maximizing the objective function given by the equations above was carried out using Powell’s optimization (Powell, 1964) as implemented in SciPy for Python with prior bounds between 0 and 1.

### Gaze Task

**Figure 2:**
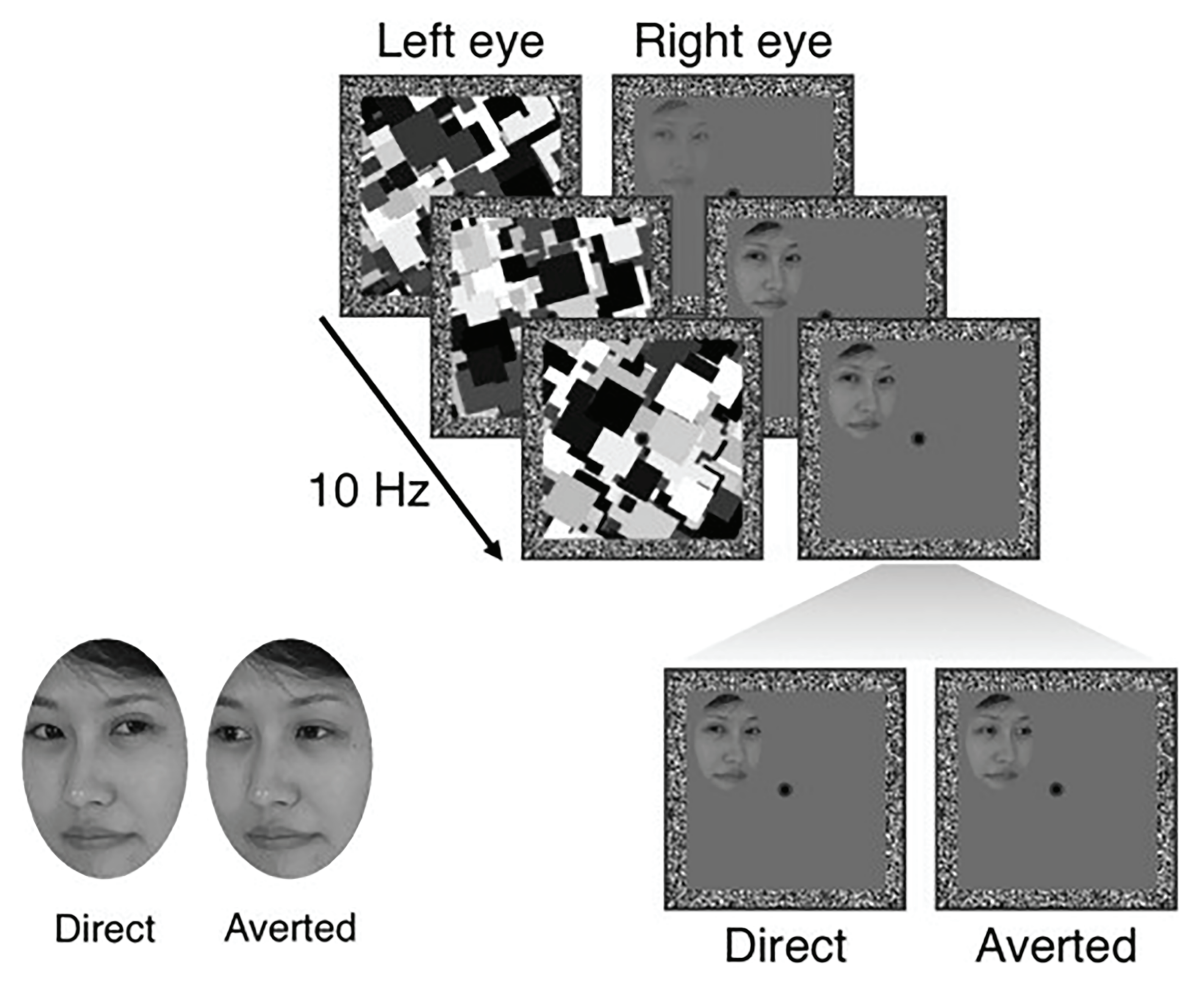
Experimental sequence of the unconscious gaze task. Both eyes of the participants received separate stimulation through a mirror stereoscope. One eye was shown the target stimulus (faces with either direct or averted gaze), whose conscious perception was suppressed by a dynamical mask shown to the other eye. Participants were instructed to indicate the localization of a face as soon as it broke through the mask, while not being told that faces differed with respect to gaze. By subtracting mean response times for averted gaze from mean response times for direct gaze, we obtained a measure of the preferential unconscious processing or prior for direct gaze. Figure adapted from Stein et al. (2011b).

To quantify the effect of individual priors for socially meaningful information on the access of visual stimuli to awareness, we used an established interocular suppression task with face stimuli that displayed either direct or averted gaze (Stein et al., 2011b; Seymour et al., 2016; Madipakkam et al., 2019). In this task, stimuli were photographs of three different female faces, each in a version with direct and averted gaze. The impression of eye gaze being either directed at or away from the observer was achieved by a shift of the pupil to the left or the right. For example, a head rotated to the right together with the pupil shifted to the left resulted in the impression of a face looking at the observer (see Figure 2, lower left). All faces were cut into oval shapes comprising a size of 3.8° × 4.5° and equalized for global contrast and luminance. Participants viewed the screen through a mirror stereoscope, which provided separate visual input to the two eyes. The participant’s head was stabilized by a chin rest at a viewing distance of 50 cm and stimuli were displayed on a 19-inch CRT monitor (resolution: 1024 × 768 Px; refresh rate: 60 Hz).

The effect of eye gaze on access of face stimuli to awareness was assessed using bCFS. Each trial began with a 2-s presentation of white frames (12.0° × 12.0°) with a gray background and a red fixation cross (Figure 2). Thereafter, high-contrast, gray scale, dynamic masks were flashed to a randomly selected eye at a frequency of 10 Hz, while simultaneously a face stimulus with either a direct or averted gaze was gradually introduced to the other eye. The contrast of the face stimulus was gradually increased from 0 to 100% within the first second from the beginning of the trial and the stimulus remained at maximum contrast until a response was made or for a maximum of 15 s. The stimuli could be presented in one of the four quadrants of the white frame (3.4° horizontal displacement from the fixation cross and 3° vertical displacement). Participants had to indicate the location of the face (i.e., the quadrant) by button press as soon as they detected a face. Importantly, participants’ task (i.e., location discrimination) was orthogonal to the condition of interest (i.e., gaze direction of the presented faces). Participants were therefore unaware of the existence of two different gaze directions. Participants completed a total of 48 trials (12 trials with direct gaze shown on the left, 12 trials with direct gaze shown on the right, 12 trials with averted gaze shown on the left, and 12 trials with averted gaze shown on the right) in a randomized order. Target variables were the response (breakthrough) times for correctly localized faces.

### Gaze Task Analysis

Three of the 39 participants were not included in the gaze task analysis, one because of technical problems and two because of the task did not work due to excessive stimulus suppression by the mask (more than 65% missed trials).

Analogously to previous studies using the same task, we compared mean breakthrough times separately for faces with direct and averted gaze. By subtracting breakthrough times for direct gaze from breakthrough times for averted gaze, we obtained a measure of the tendency toward faster access to awareness of direct gaze. In the following, we denote this measure as “direct gaze bias,” where a positive value indicates shorter breakthrough times for direct gaze relatively relative to averted gaze. As a sanity check, we first tested if this measure was significantly above zero (Bayesian one sample *t* test), e.g., whether we could replicate previous findings of a generally faster breakthrough of direct gaze. Secondly, we tested whether the degree of this direct gaze bias depended on the individual’s psychosis proneness by correlating it with CAPS and PDI scores.

### Relationships Between Psychosis Proneness, Face Bias, and Direct Gaze Bias

Statistical analyses were carried out in SPSS 27 and SciPy for Python. Psychosis proneness (CAPS and PDI scores) has been found to describe non-normal, skewed distributions in the general population samples (i.e., Peters et al., 2004; Bell et al., 2006; Stuke et al., 2017, 2018), and the distribution of the direct gaze bias was better described by uniform than by a normal distribution (Akaike information criterion for fitted uniform or normal distribution, as implemented in the scipy.stats library). Therefore, we could not assume normality of our data and analyzed the relationship between psychosis proneness and behavioral measures using rank correlations. In order to obtain Bayes factors for hypothesis testing, we first performed a rank transformation of the data and then used Bayesian correlations (default implementation in SPSS 27) to investigate relationships between delusion and hallucination proneness, face bias, and direct gaze bias.

We report correlation coefficients both with frequentist values of *p* as well as Bayes factors with the likelihood ratio between the hypothesis of no correlation, and the hypothesis of existing correlation between the tested variables [P(D|H0)/P(D|H1)]. Here, BF > 1 indicates evidence against a correlation, while BF < 1 indicate evidence for a correlation. Moreover, BF > 10 or BF < 1/10 are considered as “strong” evidence, while 3.2 < BF < 10 or 1/10 < BF < 1/3.2 indicate “substantial” evidence and BF < 3.2 or BF > 1/3.2 are viewed as evidence “barely worth mentioning” (Jeffreys, 1998).

## Results

### Participants and Psychometry

Table 1 summarizes basic demographics as well as delusion proneness (PDI scores) and hallucination proneness (CAPS scores) of the sample. In our non-clinical sample, the mean PDI-21 score was comparably high with 77.0 (42.9) as compared to 58.9 (48.0) in the non-clinical sample of the original publication of the questionnaire (Peters et al., 2004). Moreover, 12.8% (five individuals) had PDI total scores above 130, which was the mean score for the clinical sample of schizophrenia patients in the original publication. Thus, we observed a range of delusional symptoms that overlapped with the range found in samples with clinical disease.

**Table 1:** Participants’ characteristics.

Characteristic	Mean (SD)
Age	30.31 (10.06)
PDI score	76.95 (42.86)
CAPS score	106.90 (48.81)
**Characteristic**	**Absolute numbers**
Sex	Female: 19 and male: 20
Smoking	Yes: 11; no: 26; and missing information: 2
Vocation	None: 8; apprenticeship: 1; bachelor: 16; and master: 14

Similarly, the mean CAPS score we observed was comparably high with 106.9 as compared to 44.4 in the non-clinical sample in the original publication of the questionnaire (Bell et al., 2011). Here, 7.7% (three individuals) had a total score higher than 172, which was the mean of the clinical patient sample in the original study by Bell et al. (2011). Hence, our sample showed comparably high psychosis proneness with a considerable number of individuals with a degree of symptoms previously observed in clinical populations.

### Face and Gaze Task Results

**Figure 3:**
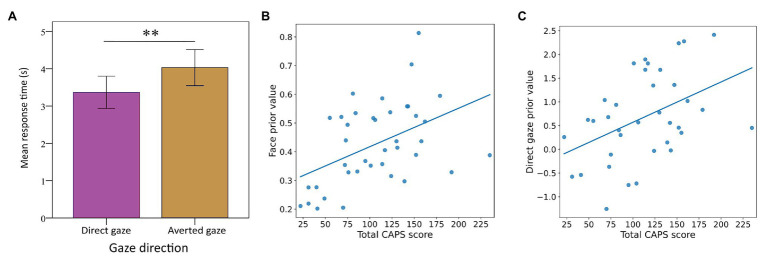
Mean response times for direct and averted gaze in the unconscious gaze task **(A)**. Consistent with previous studies, response times were significantly faster for direct gaze (*T* = −4.362, *p* < 0.001). Relationships between participants’ hallucination proneness (CAPS scores) and signal detection bias in the face task **(B)** and direct gaze bias in the unconscious gaze task **(C)**. With growing hallucination proneness, participants show an increased readiness to detect faces in noise and actual face stimuli (rho = 0.500, *p* = 0.001) as well as to unconsciously process direct gaze faster than averted gaze (rho = 0.424, *p* = 0.010). **p < 0.01.

In the face task, the mean value (SD) for the estimated face prior was 0.427 (0.143) and the sensitivity parameter 0.737 (0.080). The prior for face detection correlated with both hallucination proneness (*r* = 0.496, *p* = 0.001, *n* = 39, BF 1/20.83) and delusion proneness (*r* = 0.461, *p* = 0.003, *n* = 39, BF 1/9.43). These results suggest that psychosis proneness is associated with an increased prior for faces in a detection-in-noise task (Figure 3B).

In contrast, the sensitivity measure was not significantly related to hallucination proneness (*r* = −0.138, *p* = 0.401, *n* = 39, BF 5.65) or delusion proneness (*r* = −0.218, *p* = 0.183, *n* = 39, BF 3.33). Thus, there was no evidence for a significant association of psychosis proneness with the ability to discriminate between face and noise stimuli.

In the gaze task, breakthrough times were on average 3.385 s (1.310) for direct gaze and 4.055 s (1.465) for averted gaze. Hence, breakthrough times were significantly shorter for direct compared to averted gaze (paired *t* test, *T* = −4.362, *p* < 0.001, *n* = 36, BF = 1/20). Consistent with previous work (Stein et al., 2011b; Seymour et al., 2016), this result indicates a general direct-gaze bias for access to awareness in the whole sample (Figure 3A).

Breakthrough times were not directly correlated with hallucination or delusion proneness (all values of *p* > 0.105, all BF < 2.1). However, when we calculated the difference between breakthrough times for direct and averted gaze as a measure of the direct gaze bias, this direct gaze bias correlated significantly with hallucination proneness (*r* = 0.429, *p* = 0.008, *n* = 36, BF 1/3.83), but not significantly with delusion proneness (PDI scores, *r* = 0.297, *p* = 0.079, *n* = 36, BF 1.67). These results indicate that hallucination proneness is associated with enhanced access of direct gaze to awareness, suggesting a stronger a prior for socially relevant visual information (Figure 3C).

## Discussion

In the present work, we showed that an increased prior for faces in noisy visual stimuli was related to higher hallucination and delusion proneness and an enhanced processing of direct compared to averted invisible gaze was related to higher hallucination proneness, but not to delusion proneness. These results are largely compatible with strong prior accounts of hallucinations (Corlett et al., 2019; Horga and Abi-Dargham, 2019) that state that overly strong priors during naturally noisy perception lead to the false perception of meaning in noise, which in turn is the substrate of psychotic experiences such as hallucinations and delusions.

A Bayesian approach to experimental evidence can take into consideration not only the single study, but also integrate it with evidence from prior studies when estimating posterior probabilities of hypotheses. In our study, the Bayes factors yielded strong evidence (BF > 10; Jeffreys, 1998) for correlations between hallucination proneness and an increased face prior as well as substantial evidence (BF > 3.2) for correlations between delusion proneness and an increased face prior. Moreover, there is prior work pointing into the direction of an increased face perception bias in psychosis proneness (Partos et al., 2016). Hence, the combined evidence renders it very likely that psychosis (proneness) is related to an increased prior for face detection in noisy but visible stimuli.

The combined evidence for an increased unconscious bias for direct gaze is less clear. In the present study, we found substantial evidence (BF > 3.2) *for* a correlation between hallucination proneness and direct gaze bias. We also found evidence *against* the hypothesis of a correlation between delusion proneness and direct gaze bias, whose significance, however, was “barely worth mentioning” (BF < 3.2; Jeffreys, 1998). Prior work investigating an unconscious direct gaze bias in psychotic patients compared to healthy controls with a very similar task yielded a result that was numerically in the direction of an increased direct gaze bias in patients but fell of significance (Seymour et al., 2016). One explanation for these conflicting findings might be that a direct gaze bias was less present in the specific sample of patients investigated by Seymour et al. (2016) which was a high functioning, stable medicated chronic sample with mean illness duration of more 23 years and mild symptomatology. In this context it is noteworthy, that in our study, the direct gaze prior was specific to hallucinations (contrary to delusions), which are absent in around one third of untreated schizophrenia patients (Sartorius et al., 1986) and are significantly further reduced by antipsychotic medication (Sommer et al., 2012). Another reason might lie in slightly different properties of the CFS tasks used in this study and in the study by Seymour et al. For instance, in the task used by Seymour et al., the mask intensity was gradually decreased after target stimulus fade-in, whereas in our task version, the mask intensity did not change. Gradual fade-out of the mask may render bCFS less sensitive for the detection of individual differences (Munkler et al., 2015). Nevertheless, the mean breakthrough times as well as gaze-dependent breakthrough time differences lie in a similar range in both studies, rendering major differences in task-elicited effects unlikely. Finally, it should be noted that Seymour et al. also report increased response time differences in patients, which however did not reach significance. While it is difficult to combine these results with our present findings in a formal Bayesian analysis due to different analyses (group analyses vs. correlational analyses), interpreting these results together suggests the possibility of increased processing of direct gaze related to hallucinations, while the evidence speaks against a specific association with delusions. However, for further clarification, a follow-up experiment would be necessary to investigate the unconscious direct gaze bias in acutely psychotic patients with hallucinations.

Our finding of a relationship between hallucination proneness and an increased prior for direct gaze in a masking task is of relevance for the ongoing debate about the processing stage, at which psychosis-typical perceptual alterations take effect (Berkovitch et al., 2017). Here, our finding speaks for an involvement of unconscious processing stages. In this context, the neural correlates of these psychosis-associated unconscious processing alterations remain subject to speculation. In a first study on the neural correlates of unconscious direct gaze bias using EEG and CFS, Yokoyama et al. (2013) found increased activity on the fronto-parietal, but not on the occipital electrodes, for invisible direct gaze compared to averted gaze. Using fMRI, Madipakkam et al. (2015) found decreased activation of the fusiform face area, superior temporal sulcus, amygdala, and intraparietal sulcus for invisible direct gaze and concluded that in these regions, lower levels of neural activity are sufficient to give rise to awareness for direct than for averted gaze. Both findings speak for a differential involvement of higher-level processing stages in the processing of invisible direct compared to averted gaze. Studies investigating psychosis-associated changes in the neural underpinnings of unconscious processing of gaze remain to be conducted.

It should be noted that, in our current work “prior” does not refer to experimentally manipulated information, but to an implicit expectation of socially meaningful signals (faces and direct gaze) in noisy and ambiguous stimuli. This is in contrast to a body of previous work, where prior information was experimentally varied and had to be balanced against (potentially contradictory) sensory information. Here, relationships between psychotic experiences and prior usage were less consistent. In some experiments, there was an increased prior usage with growing psychosis proneness (Corlett et al., 2019 for a review), which is in line with our current results, while other studies showed even porting *decreased* prior usage with growing psychosis proneness (Jardri et al., 2017; Stuke et al., 2018). This inconsistency goes well with the emerging understanding that different kinds of priors may be differentially affected in psychosis, and that alterations in perceptual inference go beyond a simple over- or underweighting of priors. In short, it is proposed, that strong “high-level” belief priors might compensate for weak “low-level” sensory priors (for detailed discussions, see Schmack et al., 2013; Sterzer et al., 2018; Heinz et al., 2019). In this framework, our results are consistent with the hypothesis of stronger high-level priors (i.e., an increased prior probability for the presence of faces and direct gaze in hallucination-prone individuals).

The present results raise the question of what processes might underlie the bias toward detecting socially relevant information we observed in hallucination proneness. In theory, three possibilities are conceivable here: first, the increased tendency to perceive faces and direct gaze could be based on a *general information processing* bias such as overhasty decision-making. However, this possibility has not been confirmed by previous work that failed to show a connection between jumping to conclusions in a non-perceptual task on the one hand, and hallucinations or jumping to erroneous perceptions in a perceptual task on the other (Bristow et al., 2014). An increased tendency to perceive faces and direct gaze could, second, represent specific changes in the *processing of sensory information*, or, third, even more specific changes in the *processing of socially relevant sensory information*. In our view, these latter two possibilities cannot be distinguished with certainty at the moment. Partos et al. (2016) reported a bias toward perceiving signals in visual noise using analyses that combined non-socially relevant stimuli (natural scences) and potentially socially relevant stimuli (cartoons with mostly anthropomorphised animals). In a second experiment, the content participants perceived in visual noise could be entered freely by the participants and participants reported “36% contained human faces or facial features, 25% animals or mythical creatures, 20% humanoid figures, 15% natural objects or scenes, and 4% other” (Partos et al., 2016). These figures suggest a predominance of socially relevant stimuli in the erroneous percepts, but do not rule out that this might be due to changes in the processing in sensory information in general. Bristow et al. (2014) expanded a classic auditory experiment (Bentall and Slade, 1985) into the visual domain. Here, the target stimulus was the word “who,” which had been denoted as socially relevant in the original paper (“The word ‘Who’ was chosen because it is short, common and because it was considered that the word used should make some reference to the subject. Hallucinating individuals commonly hear their voices speaking to themselves or commenting about their own actions”; Bentall and Slade, 1985). Participants with current hallucinations showed an increased bias for perceiving this written or spoken word in visual and auditory noise, respectively, in line with an altered processing of socially relevant sensory information in both the visual and auditory domain. However, because only socially relevant sensory stimuli were used, these findings do not rule out a more generic alteration in the processing of sensory information. Hence, future work using a detection task similar to ours but with socially irrelevant stimuli as an additional control condition will be necessary to pinpoint whether the visual processing alterations related to hallucinations represent generic sensory processing alterations, or are specific to socially relevant stimuli.

False alarms in detection tasks (perception of meaning from noise stimuli) have an intuitive “face validity” as experimental hallucination markers. Similarly, the preferential unconscious processing of direct gaze directly relates to the psychotic feeling of being stared at in public. Hence, as opposed to other common markers for psychosis proneness (e.g., a reduced EEG mismatch negativity; Naatanen et al., 2015; Erickson et al., 2016) and cognitive biases, such as jumping-to-conclusions (Dudley et al., 2016), the two tasks used here have an immediate connection to the phenomenology of psychosis and might serve as symptom-related markers for the severity of psychotic experiences. It might be a worthwhile endeavor to investigate the predictive power of these markers in further research. In clinical settings, an early response of psychosis-related markers after initiation of antipsychotic treatment might help to predict following treatment response. In preclinical research, similar detection-in-noise tasks might help to assess effects of pro- or anti-psychotic interventions in animal models. In any case, the development of suited experimental markers to monitor and predict the effect of psychosis-targeting interventions remains an important cornerstone for progressing our still limited understanding and treatment options for psychotic disorders.

A limitation of the present results is that we investigated correlates of psychosis proneness in healthy individuals only. While the psychosis continuum framework described in the introduction suggests that the relationships found are meaningful for clinical manifestations of psychosis, a follow-up study involving psychotic patients would be required for confirmation.

In summary, our results speak to an overly strong prior for socially meaningful information in people with psychotic experiences that extends beyond the domain of auditory perception and might also affect early unconscious stages of sensory processing.

## Data Availability Statement

The raw data supporting the conclusions of this article will be made available by the authors, without undue reservation.

## Ethics Statement

The studies involving human participants were reviewed and approved by Ethical Committee of the Charité, Universitätsmedizin Berlin. The patients/participants provided their written informed consent to participate in this study.

## Author Contributions

HS, KS, PS, and VW developed the experimental design. EK carried out the experiment. HS, EK, and KS performed the data analysis and wrote the manuscript. All authors contributed to the article and approved the submitted version.

### Conflict of Interest

The authors declare that the research was conducted in the absence of any commercial or financial relationships that could be construed as a potential conflict of interest.

## References

[ref1] Adams R. A. Stephan K. E. Brown H. R. Frith C. D. Friston K. J. (2013). The computational anatomy of psychosis. Front. Psychol. 4:47. 10.3389/fpsyt.2013.00047, PMID: 23750138 PMC3667557

[ref2] Akechi H. Stein T. Senju A. Kikuchi Y. Tojo Y. Osanai H.. (2014). Absence of preferential unconscious processing of eye contact in adolescents with autism spectrum disorder. Autism Res. 7, 590–597. 10.1002/aur.1397, PMID: 24962761

[ref3] Alderson-Day B. Lima C. F. Evans S. Krishnan S. Shanmugalingam P. Fernyhough C.. (2017). Distinct processing of ambiguous speech in people with non-clinical auditory verbal hallucinations. Brain 140, 2475–2489. 10.1093/brain/awx206, PMID: 29050393

[ref4] Barrantes-Vidal N. Grant P. Kwapil T. R. (2015). The role of schizotypy in the study of the etiology of schizophrenia spectrum disorders. Schizophr. Bull. 41(Suppl 2), S408–S416. 10.1093/schbul/sbu191, PMID: 25810055 PMC4373635

[ref5] Bell V. Halligan P. W. Ellis H. D. (2006). The Cardiff anomalous perceptions scale (CAPS): a new validated measure of anomalous perceptual experience. Schizophr. Bull. 32, 366–377. 10.1093/schbul/sbj014, PMID: 16237200 PMC2632213

[ref6] Bell V. Halligan P. W. Pugh K. Freeman D. (2011). Correlates of perceptual distortions in clinical and non-clinical populations using the Cardiff anomalous perceptions scale (CAPS): associations with anxiety and depression and a re-validation using a representative population sample. Psychiatry Res. 189, 451–457. 10.1016/j.psychres.2011.05.025, PMID: 21703692

[ref7] Bentall R. P. Slade P. D. (1985). Reality testing and auditory hallucinations: a signal detection analysis. Br. J. Clin. Psychol. 24, 159–169. 10.1111/j.2044-8260.1985.tb01331.x, PMID: 4052663

[ref8] Berkovitch L. Dehaene S. Gaillard R. (2017). Disruption of conscious access in schizophrenia. Trends Cogn. Sci. 21, 878–892. 10.1016/j.tics.2017.08.006, PMID: 28967533

[ref9] Bristow E. Tabraham P. Smedley N. Ward T. Peters E. (2014). Jumping to perceptions and to conclusions: specificity to hallucinations and delusions. Schizophr. Res. 154, 68–72. 10.1016/j.schres.2014.02.004, PMID: 24581551

[ref10] Chapman L. J. Chapman J. P. Kwapil T. R. Eckblad M. Zinser M. C. (1994). Putatively psychosis-prone subjects 10 years later. J. Abnorm. Psychol. 103, 171–183. 10.1037/0021-843X.103.2.171, PMID: 8040487

[ref11] Corlett P. R. Frith C. D. Fletcher P. C. (2009). From drugs to deprivation: a Bayesian framework for understanding models of psychosis. Psychopharmacology 206, 515–530. 10.1007/s00213-009-1561-0, PMID: 19475401 PMC2755113

[ref12] Corlett P. R. Horga G. Fletcher P. C. Alderson-Day B. Schmack K. Powers A. R. 3rd (2019). Hallucinations and strong priors. Trends Cogn. Sci. 23, 114–127. 10.1016/j.tics.2018.12.001, PMID: 30583945 PMC6368358

[ref13] DeRosse P. Karlsgodt K. H. (2015). Examining the psychosis continuum. Curr. Behav. Neurosci. Rep. 2, 80–89. 10.1007/s40473-015-0040-7, PMID: 26052479 PMC4454466

[ref14] Dudley R. Taylor P. Wickham S. Hutton P. (2016). Psychosis, delusions and the “jumping to conclusions” reasoning bias: a systematic review and meta-analysis. Schizophr. Bull. 42, 652–665. 10.1093/schbul/sbv150, PMID: 26519952 PMC4838082

[ref15] Erickson M. A. Ruffle A. Gold J. M. (2016). A meta-analysis of mismatch negativity in schizophrenia: from clinical risk to disease specificity and progression. Biol. Psychiatry 79, 980–987. 10.1016/j.biopsych.2015.08.025, PMID: 26444073 PMC4775447

[ref16] Fanous A. Gardner C. Walsh D. Kendler K. S. (2001). Relationship between positive and negative symptoms of schizophrenia and schizotypal symptoms in nonpsychotic relatives. Arch. Gen. Psychiatry 58, 669–673. 10.1001/archpsyc.58.7.669, PMID: 11448374

[ref17] Fletcher P. C. Frith C. D. (2009). Perceiving is believing: a Bayesian approach to explaining the positive symptoms of schizophrenia. Nat. Rev. Neurosci. 10, 48–58. 10.1038/nrn2536, PMID: 19050712

[ref18] Franck N. Montoute T. Labruyere N. Tiberghien G. Marie-Cardine M. Dalery J.. (2002). Gaze direction determination in schizophrenia. Schizophr. Res. 56, 225–234. 10.1016/S0920-9964(01)00263-8, PMID: 12072171

[ref19] Galdos M. Simons C. Fernandez-Rivas A. Wichers M. Peralta C. Lataster T.. (2011). Affectively salient meaning in random noise: a task sensitive to psychosis liability. Schizophr. Bull. 37, 1179–1186. 10.1093/schbul/sbq029, PMID: 20360211 PMC3196950

[ref20] Hanssen M. Bak M. Bijl R. Vollebergh W. van Os J. (2005). The incidence and outcome of subclinical psychotic experiences in the general population. Br. J. Clin. Psychol. 44, 181–191. 10.1348/014466505X29611, PMID: 16004653

[ref21] Heinz A. Murray G. K. Schlagenhauf F. Sterzer P. Grace A. A. Waltz J. A. (2019). Towards a unifying cognitive, neurophysiological, and computational neuroscience account of schizophrenia. Schizophr. Bull. 45, 1092–1100. 10.1093/schbul/sby154, PMID: 30388260 PMC6737474

[ref22] Hoffman R. E. Woods S. W. Hawkins K. A. Pittman B. Tohen M. Preda A.. (2007). Extracting spurious messages from noise and risk of schizophrenia-spectrum disorders in a prodromal population. Br. J. Psychiatry 191, 355–356. 10.1192/bjp.bp.106.031195, PMID: 17906248

[ref23] Hooker C. Park S. (2005). You must be looking at me: the nature of gaze perception in schizophrenia patients. Cogn. Neuropsychol. 10, 327–345. 10.1080/13546800444000083, PMID: 16571465

[ref24] Horga G. Abi-Dargham A. (2019). An integrative framework for perceptual disturbances in psychosis. Nat. Rev. Neurosci. 20, 763–778. 10.1038/s41583-019-0234-1, PMID: 31712782

[ref25] Jardri R. Duverne S. Litvinova A. S. Deneve S. (2017). Experimental evidence for circular inference in schizophrenia. Nat. Commun. 8:14218. 10.1038/ncomms14218, PMID: 28139642 PMC5290312

[ref26] Jeffreys H. (1998). The theory of probability. Oxford: Oxford University Press.

[ref27] Jiang Y. Costello P. He S. (2007). Processing of invisible stimuli: advantage of upright faces and recognizable words in overcoming interocular suppression. Psychol. Sci. 18, 349–355. 10.1111/j.1467-9280.2007.01902.x, PMID: 17470261

[ref28] Kendler K. S. McGuire M. Gruenberg A. M. O'Hare A. Spellman M. Walsh D. (1993). The roscommon family study. III. Schizophrenia-related personality disorders in relatives. Arch. Gen. Psychiatry 50, 781–788. 10.1001/archpsyc.1993.01820220033004, PMID: 8215802

[ref29] Linscott R. J. van Os J. (2013). An updated and conservative systematic review and meta-analysis of epidemiological evidence on psychotic experiences in children and adults: on the pathway from proneness to persistence to dimensional expression across mental disorders. Psychol. Med. 43, 1133–1149. 10.1017/S0033291712001626, PMID: 22850401

[ref30] Madipakkam A. R. Rothkirch M. Dziobek I. Sterzer P. (2019). Access to awareness of direct gaze is related to autistic traits. Psychol. Med. 49, 980–986. 10.1017/S0033291718001630, PMID: 29947310

[ref31] Madipakkam A. R. Rothkirch M. Guggenmos M. Heinz A. Sterzer P. (2015). Gaze direction modulates the relation between neural responses to faces and visual awareness. J. Neurosci. 35, 13287–13299. 10.1523/JNEUROSCI.0815-15.2015, PMID: 26424878 PMC6605480

[ref100] Minear M. Park D. C. (2004). A lifespan database of adult facial stimuli. Behav. Res. Methods. Instrum. Comput. 36, 630–633. 10.3758/bf03206543, PMID: 15641408

[ref32] Munkler P. Rothkirch M. Dalati Y. Schmack K. Sterzer P. (2015). Biased recognition of facial affect in patients with major depressive disorder reflects clinical state. PLoS One 10:e0129863. 10.1371/journal.pone.0129863, PMID: 26039710 PMC4454562

[ref33] Naatanen R. Shiga T. Asano S. Yabe H. (2015). Mismatch negativity (MMN) deficiency: a break-through biomarker in predicting psychosis onset. Int. J. Psychophysiol. 95, 338–344. 10.1016/j.ijpsycho.2014.12.012, PMID: 25562834

[ref34] Partos T. R. Cropper S. J. Rawlings D. (2016). You don't see what I see: individual differences in the perception of meaning from visual stimuli. PLoS One 11:e0150615. 10.1371/journal.pone.0150615, PMID: 26954696 PMC4783041

[ref35] Peters E. Joseph S. Day S. Garety P. (2004). Measuring delusional ideation: the 21-item Peters et al. delusions inventory (PDI). Schizophr. Bull. 30, 1005–1022. 10.1093/oxfordjournals.schbul.a007116, PMID: 15954204

[ref36] Powell M. (1964). An efficient method for finding the minimum of a function of several variables without calculating derivatives. Comput. J. 7, 155–162. 10.1093/comjnl/7.2.155

[ref37] Powers A. R. Mathys C. Corlett P. R. (2017). Pavlovian conditioning-induced hallucinations result from overweighting of perceptual priors. Science 357, 596–600. 10.1126/science.aan3458, PMID: 28798131 PMC5802347

[ref38] Rosse R. B. Kendrick K. Wyatt R. J. Isaac A. Deutsch S. I. (1994). Gaze discrimination in patients with schizophrenia: preliminary report. Am. J. Psychiatry 151, 919–921. 10.1176/ajp.151.6.919, PMID: 8185005

[ref39] Sartorius N. Jablensky A. Korten A. Ernberg G. Anker M. Cooper J. E.. (1986). Early manifestations and first-contact incidence of schizophrenia in different cultures. A preliminary report on the initial evaluation phase of the WHO collaborative study on determinants of outcome of severe mental disorders. Psychol. Med. 16, 909–928. 10.1017/S0033291700011910, PMID: 3493497

[ref40] Schmack K. Burk J. Haynes J. D. Sterzer P. (2016). Predicting subjective affective salience from cortical responses to invisible object stimuli. Cereb. Cortex 26, 3453–3460. 10.1093/cercor/bhv174, PMID: 26232987

[ref41] Schmack K. Gomez-Carrillo de Castro A. Rothkirch M. Sekutowicz M. Rossler H. Haynes J. D.. (2013). Delusions and the role of beliefs in perceptual inference. J. Neurosci. 33, 13701–13712. 10.1523/JNEUROSCI.1778-13.2013, PMID: 23966692 PMC6618656

[ref42] Seymour K. Rhodes G. Stein T. Langdon R. (2016). Intact unconscious processing of eye contact in schizophrenia. Schizophr. Res. Cogn. 3, 15–19. 10.1016/j.scog.2015.11.001, PMID: 28740803 PMC5506706

[ref43] Shevlin M. McElroy E. Bentall R. P. Reininghaus U. Murphy J. (2017). The psychosis continuum: testing a bifactor model of psychosis in a general population sample. Schizophr. Bull. 43, 133–141. 10.1093/schbul/sbw067, PMID: 27220965 PMC5216850

[ref44] Sommer I. E. Slotema C. W. Daskalakis Z. J. Derks E. M. Blom J. D. van der Gaag M. (2012). The treatment of hallucinations in schizophrenia spectrum disorders. Schizophr. Bull. 38, 704–714. 10.1093/schbul/sbs034, PMID: 22368234 PMC3577047

[ref45] Stanislaw H. Todorov N. (1999). Calculation of signal detection theory measures. Behav. Res. Methods Instrum. Comput. 31, 137–149. 10.3758/BF03207704, PMID: 10495845

[ref46] Stein T. Hebart M. N. Sterzer P. (2011a). Breaking continuous flash suppression: a new measure of unconscious processing during interocular suppression? Front. Hum. Neurosci. 5:167. 10.3389/fnhum.2011.00167, PMID: 22194718 PMC3243089

[ref47] Stein T. Senju A. Peelen M. V. Sterzer P. (2011b). Eye contact facilitates awareness of faces during interocular suppression. Cognition 119, 307–311. 10.1016/j.cognition.2011.01.008, PMID: 21316650 PMC3796336

[ref48] Stein T. Sterzer P. (2014). Unconscious processing under interocular suppression: getting the right measure. Front. Psychol. 5:387. 10.3389/fpsyg.2014.00387, PMID: 24834061 PMC4018522

[ref49] Sterzer P. Adams R. A. Fletcher P. Frith C. Lawrie S. M. Muckli L.. (2018). The predictive coding account of psychosis. Biol. Psychiatry 84, 634–643. 10.1016/j.biopsych.2018.05.015, PMID: 30007575 PMC6169400

[ref50] Sterzer P. Hilgenfeldt T. Freudenberg P. Bermpohl F. Adli M. (2011). Access of emotional information to visual awareness in patients with major depressive disorder. Psychol. Med. 41, 1615–1624. 10.1017/S0033291710002540, PMID: 21208495

[ref105] Stuke H. Kress E. Weilnhammer V. A. Sterzer P. Schmack K. (2018). Overly strong priors for socially meaningful visual signals in psychosis proneness. bioRxiv, 473421 [Preprint]. 10.1101/473421, PMID: 33897518 PMC8061414

[ref51] Stuke H. Stuke H. Weilnhammer V. A. Schmack K. (2017). Psychotic experiences and overhasty inferences are related to maladaptive learning. PLoS Comput. Biol. 13:e1005328. 10.1371/journal.pcbi.1005328, PMID: 28107344 PMC5249047

[ref52] Stuke H. Weilnhammer V. A. Sterzer P. Schmack K. (2018). Delusion proneness is linked to a reduced usage of prior beliefs in perceptual decisions. Schizophr. Bull. 45, 80–86. 10.1093/schbul/sbx189, PMID: 29365194 PMC6293222

[ref53] Tienari P. Wynne L. C. Laksy K. Moring J. Nieminen P. Sorri A.. (2003). Genetic boundaries of the schizophrenia spectrum: evidence from the Finnish adoptive family study of schizophrenia. Am. J. Psychiatry 160, 1587–1594. 10.1176/appi.ajp.160.9.1587, PMID: 12944332

[ref54] Tso I. F. Mui M. L. Taylor S. F. Deldin P. J. (2012). Eye-contact perception in schizophrenia: relationship with symptoms and socioemotional functioning. J. Abnorm. Psychol. 121, 616–627. 10.1037/a0026596, PMID: 22250658

[ref55] Tsuchiya N. Koch C. (2005). Continuous flash suppression reduces negative afterimages. Nat. Neurosci. 8, 1096–1101. 10.1038/nn1500, PMID: 15995700

[ref56] van Os J. Linscott R. J. Myin-Germeys I. Delespaul P. Krabbendam L. (2009). A systematic review and meta-analysis of the psychosis continuum: evidence for a psychosis proneness-persistence-impairment model of psychotic disorder. Psychol. Med. 39, 179–195. 10.1017/S0033291708003814, PMID: 18606047

[ref57] Vercammen A. de Haan E. H. Aleman A. (2008). Hearing a voice in the noise: auditory hallucinations and speech perception. Psychol. Med. 38, 1177–1184. 10.1017/S0033291707002437, PMID: 18076771

[ref58] Welham J. Scott J. Williams G. Najman J. Bor W. O'Callaghan M.. (2009). Emotional and behavioural antecedents of young adults who screen positive for non-affective psychosis: a 21-year birth cohort study. Psychol. Med. 39, 625–634. 10.1017/S0033291708003760, PMID: 18606046

[ref59] Yokoyama T. Noguchi Y. Kita S. (2013). Unconscious processing of direct gaze: evidence from an ERP study. Neuropsychologia 51, 1161–1168. 10.1016/j.neuropsychologia.2013.04.002, PMID: 23603242

